# Camphor Medication

**Published:** 1874-05

**Authors:** 


### CAMPHOR MEDICATION.

Camphor is a poison, and yet it is largely used in many families for alleviating pain and curing sores. It is a nervous irritant. If taken in small doses, it acts like alcohol and opium.

If in large quantities, it excites the nervous system even to the extent of camphor spasms and death.

Camphor also acts as an irritant on the mucous membrane of the stomach, leading to constipation and ulceration; on these accounts it should not be used without the advice of a physician. Families easily get into the habit of running to the camphor bottle for every trifling ailment, until after a while insidious maladies break out whose origin is little suspected.

